# Supplementary material for: Aggressiveness and Patulin Production in Penicillium expansum Multidrug Resistant Strains with Different Expression Levels of MFS and ABC Transporters, in the Presence or Absence of Fludioxonil
Source: Plants (Basel). 2023 Mar 21;12(6):1398. doi: 10.3390/plants12061398 (PMC10056477; doi:10.3390/plants12061398)
Supplement: Supplementary file 1 [file plants-12-01398-s001.zip › plants-2183772-supplementary.pdf]

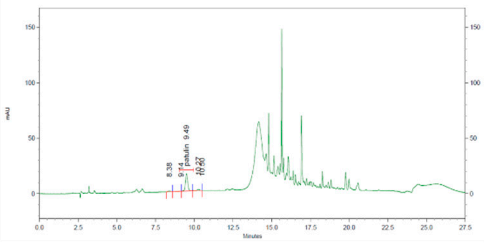

Sample: isolate ZRD13, *in vivo*, untreated fruit

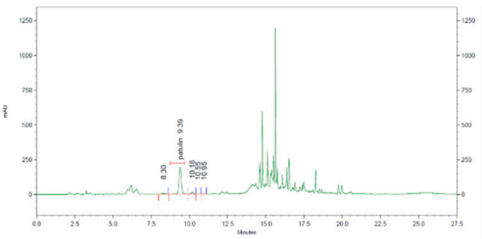

Sample: isolate HGS16, *in vivo*, untreated fruit

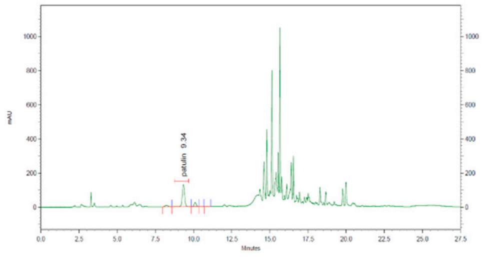

Sample: isolate ZRD13, *in vivo*, fludioxonil-treated fruit

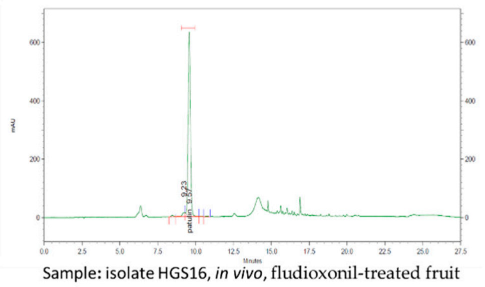

Sample: isolate HGS16, *in vivo*, fludioxonil-treated fruit

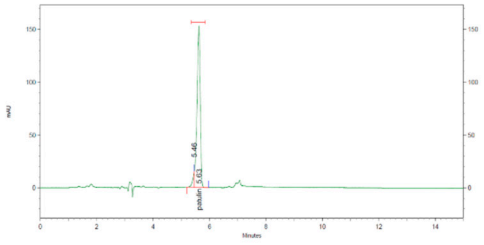

Sample: isolate ZRD13, *in vitro*, control medium

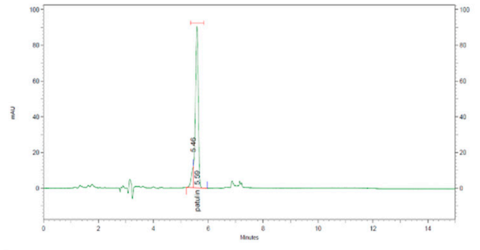

Sample: isolate HGS16, *in vitro*, control medium

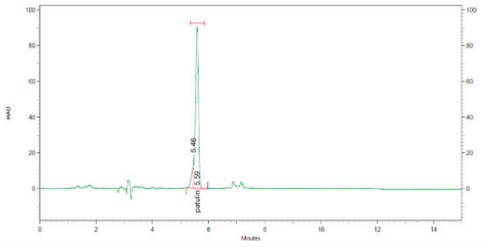

Sample: isolate ZRD13, *in vitro*, fludioxonil-amended medium

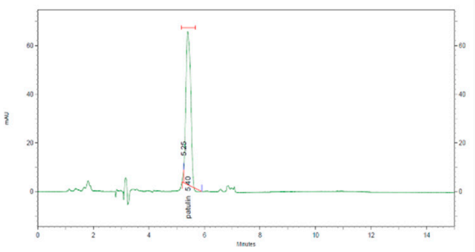

Sample: isolate HGS16, *in vitro*, fludioxonil-amended medium

Figure S1. HPLC - DAD chromatograms of mycotoxin patulin from the analysis of *in vitro* and *in vivo* samples.

Table S1. Linear gradient conditions of the solvents concentration which used for patulin chromatographic analysis.

Time (min)	% mobile phase A*	% mobile phase B
0	95	5
10	90	10
15	10	90
20	10	90
22,5	95	5
27,5	95	5

* Mobile phase A consisted of 0.1% acetic acid in water and mobile phase B of 0.1% acetic acid in acetonitrile.

Table S2. Oligonucleotides used in this study to induct of patulin biosynthesis related genes *in vitro* and *in vivo* samples with qRT-PCR.

**Primer name**	**Primer sequence (5′ – 3′)**	**Target gene**	**Related mechanism**	**Expected size (bp)**	**Source**	**Accession Number**
patC-F	CGAAGGCCGCAGCTTCTTCA	*patC*	MFS transporter	175 bp	This study	KF_899892.1
patC-R	AGCAATGGTGAGGACAGCGG					
patM-F	CCCGCACCACAGATGCAAGT	*patM*	cytochrome P450	177 bp	This study	
patM-R	TCCGAGGCACTGCACTGGTA					
patH-F	GGAGGTGATGGAAACCGGCG	*patH*	ABC transporter	177 bp	This study	
patH-R	GTGGCCATCTTCCCGTCGTT					
b-tub F	AGGCCAGCGGTGACAAGTACG	*b -tubulin*	-	80 bp	Samaras et al., 2020	AY_674400.1
b-tub R	ACAGCGTCCATGGTACCGGG					
